# Trends in Increased Infection Risk Deceased Organ Donors. A Two-Centre Retrospective Study

**DOI:** 10.1177/20543581261463456

**Published:** 2026-06-24

**Authors:** Oscar A. Fernández‐García, Justin Co, Tanya Suandork, Murdoch Leeies, Karen Elizabeth Doucette

**Affiliations:** 1 Department of Medicine, Division of Infectious Diseases, 3158 University of Alberta, Edmonton, Alberta, Canada; 2 Department of Emergency Medicine, University of Manitoba, Winnipeg, Manitoba, Canada

**Keywords:** donor screening, hepatitis B, hepatitis C, HIV, donor derived infection

## Abstract

**Background:**

Canadian regulatory standards require the label increased risk donor (IRD) be applied to donors with risk factors for hepatitis B (HBV), hepatitis C (HCV) and human immunodeficiency virus infection (HIV). This study aimed to assess trends in the rate of IRD and specific donor risk factors over time as well as the association with donor screening results for HBV, HCV and HIV.

**Methods:**

This was a retrospective study of deceased organ donors at 2 Canadian centers (University of Alberta Hospital and Transplant Manitoba) encompassing an overlapping period between 2013 and 2022. The proportion of IRD donations over time were analyzed with trend analysis. Trends for risk factors and their association with donor screening results were explored.

**Results:**

Overall, 332 of 1491 (22.3%) donors were categorized as IRD. A significant rising trend of IRD donors was documented for Alberta. Any donor drug use and overdose as cause of death increased during the study period and were strongly associated with HCV infection (OR 18.03 (6.34-51.2) p<0.001 and OR 6.48 (3.32-12.65) p<0.001). Donors classified as men who had sex with men (MSM) did not have an increased risk of HBV, HCV or HIV. Answers to some questions on the donor risk history were “I don’t know” in up to 20% of specific questionnaire items.

**Conclusions:**

Our study demonstrates a rising proportion of IRD organ donations in 2 Canadian transplant centers. The regulatory requirements include variables not associated with HBV/HCV/HIV and do not capture others associated with risk in this cohort. The proportion of “I don’t know” responses on the donor risk assessment highlights its limitations. This data may inform donor risk assessment in the Canadian setting and highlights the changing epidemiology of HBV/HCV/HIV in organ donors.

## Introduction

Solid organ transplantation (SOT) risks transmission of infectious diseases. Recommendations for infectious disease screening in organ donors have been produced by a number of professional societies.^1,2^ Health Canada and the Canadian Standards Association (CSA) provide the regulatory standards to label an organ safe for transplantation in Canada.^3,4^ The label Increased Risk Donor (IRD) must be applied when donors are determined to have had risk factors for hepatitis B virus (HBV), hepatitis C virus (HCV), and human immunodeficiency virus (HIV) infection. These risk factors are outlined in Annex E of the Canadian Standards Association (CSA) Cells, Tissues, and Organs for Transplant: General requirements (Table S1).^4^ Adjudication of these risk factors is done through a questionnaire administered to the donor’s next of kin. If the next of kin refers that a donor had any of the risk behaviours the donor is labeled IRD. “I don’t know” responses are not considered to warrant IRD designation.

Non-IRD donors are required to be screened for HBV, HCV and HIV with serological assays and recipients are not required to have posttransplant screening for HBV, HCV or HIV. Labeling a donor as IRD requires more sensitive screening using nucleic acid amplification testing (NAT). It also mandates additional recipient informed consent and posttransplant screening of the recipient for HBV, HCV and HIV infection using NAT.

Although an increase in the proportion of IRD donors has been documented in other jurisdictions, Canadian data are lacking.^5^ The aim of this project was to describe and analyze trends regarding deceased donor risk factors for bloodborne infections to inform evidence-based policy.

We used two independent datasets from two Canadian provinces’ organ donation organizations (OPOs) for this retrospective study.

The primary outcome was to determine the percentage of donors categorized as IRD over time in both provinces.

The following secondary outcomes were explored:

1. The percentage of “I don’t know” answers to risk behaviour questionnaire items.
2. A description of trends in donor risk factors for HBV, HCV and HIV infection.
3. Assess associations between risk factors and donor screening results for HBV, HCV and HIV,
4. Assess association between variables not contained in Annex E and donor screening results for HBV, HCV and HIV.

## Methods

Data sets comprised deceased organ donors who donated organs at the University of Alberta Hospital multiorgan transplant program between January 1, 2013, and December 31, 2022. Through a previous project, the Authors had access to a convenience dataset of deceased organ donors from Manitoba encompassing the years 2015-2020.^6^ All deceased organ donors who donated organs during the specified periods were included; living donors were excluded. CSA Standards require that all questions regarding behavioural risk factors in Annex E are asked to the next of kin of the deceased donor and that serology and NAT be completed for HBV, HCV and HIV in all IRD donors. As such, there were no missing data in the usual sense. Missing data consisted of “I don’t know” responses to what are framed as yes or no behavioural risk questions and was analysed as a secondary outcome.

Donor charts from Give Life Alberta and Transplant Manitoba, the OPOs serving their respective transplant programs were reviewed. Cause of death, responses to questionnaire items and donor viral screening results were recorded. Donor IRD status was classified based on responses to questionnaire items addressing the risk factors outlined in Annex E of the Canadian Standards Association (CSA) Cells, Tissues, and Organs for Transplant: General requirements (Table S1). Different coding of risk factors at collection by clinical teams precluded complete merging of the two cohorts. The donor cohort from University of Alberta was used to explore the secondary outcomes.

This study was approved by the University of Alberta Research Ethics Board (Pro00128376) and the University of Manitoba Ethics Board (HS26896) with a waiver for informed consent.

## Analysis

Categorical variables were expressed as percentages; continuous variables were expressed as median and interquartile range. Trends in proportion of IRD donors are presented for both centres. Mann-Kendall test and Sen’s slope were used for trend analysis. Odds ratios for HBV and HCV donor infection were calculated using bivariate logistic regression. Given that very few donors had detectable HBV viral load or a positive hepatitis B surface antigen (HBsAg), HBV analysis was limited to association with antibody to hepatitis B core antigen (anti-HBc). When performing multivariate analysis, we used logistic regression with backwards elimination of variables to determine the final variables to be included in the model. Variables with p>0.10 were eliminated unless required for adjustment (age and sex). Stata version 18 and RStudio-4.4.2 were used for analysis.

## Results

During the study period 1375 deceased donors donated to the University of Alberta and 116 to the Manitoba Transplant programs. Donor characteristics and prevalence of HBV, HCV and HIV infection are shown in Table 1.

**Table 1.** Donor Characteristics, HBV: Hepatitis B Virus, HBsAg: Hepatitis B Surface Antigen, Anti-HBc: Hepatitis B Core Antibody, HCV: Hepatitis C Virus, HIV: Human Immunodeficiency virus, Ab: Antibody, NAT: Nucleic Acid Amplification Test

Variable	Alberta N=1375 (%)	Manitoba N=116 (%)
Donor age	41 (27-55)	41 (25-51)
Male sex	853 (62%)	92(70%)
Increased risk donor	310 (22.5%)	22 (19%)
HBV NAT positive	3 (0.22%)	0
HBsAg positive	2 (0.15%)	0
Anti-HBc positive	48 (4.39%)	0
HCV NAT positive	30 (2.18%)	0
Anti-HCV positive	51 (3.71%)	3 (2.5%)
HIV NAT positive	0	0
HIV Ab positive	0	0

Over the study period 310 (22.5%) donors in the Alberta program met IRD criteria. In Manitoba 22 (19%) donors were categorized as IRD.

Infection by HBV and HCV were exclusively documented in the IRD population; no organs were procured from individuals with HIV infection.

No donors were positive for both anti-HCV and HBsAg. Seven donors were positive for both HCV and anti-HBc, 4 donors had a detectable HCV viral load and anti-HBc.

**Figure 1.**
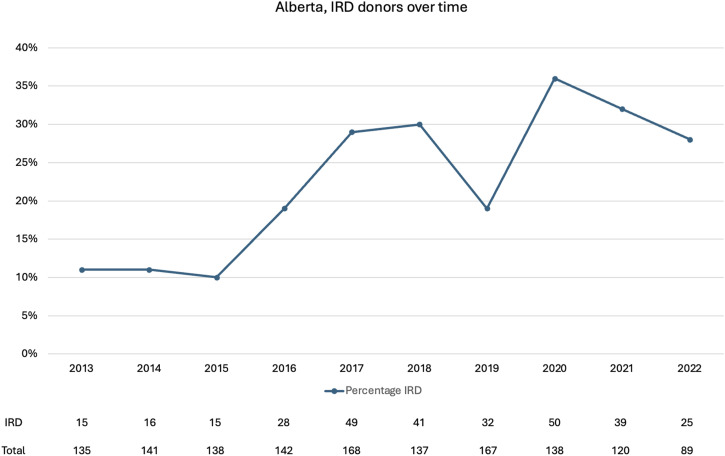
Percentage of increased risk donors over time in Alberta. IRD: Increased risk donor

**Figure 2.**
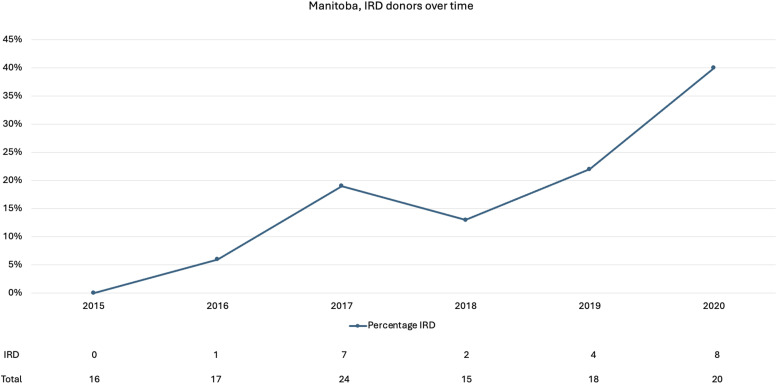
Percentage of increased risk donors over time in Manitoba. IRD: Increased risk donor

Figures 1 and 2 depict the percentage of IRD donors over time for both provinces. Man-Kendall test demonstrated a significant increasing trend (τ=0.56, p=0.0304, Sen’s slope: *Q*_*i*_=2.6 [0.6-4.25]) of IRD donors in Alberta. An increasing proportion of IRD donors was noted in Manitoba however the trend was not statistically significant (τ=0.73, p=0.0602, *Q*_*i*_=6 [-3.5 to 18]). The percentage of “I don’t know” responses to the screening questionnaire by risk factor are reported in Table 2. In Manitoba “I don’t know” responses are routinely coded as affirmative, so data is presented only for Alberta.

**Table 2.** Unknown Status of Risk Behaviours Considered “Increased Risk Donor” Criteria in Alberta. MSM: Man Who had Sex With a Man, HBV: Hepatitis B Virus, HCV: Hepatitis C Virus, HIV: Human Immunodeficiency Virus

Risk behaviour	“I do not know” responses to specific donor risk factors. N=1375 (%)
Non-medical injection drug use	69 (5.02%)
MSM	43 (3.3%)
Sex in exchange for drugs or money	106 (7.7%)
Sex with an individual from the above risk categories	274 (19.9%)
Sex with an individual with suspected HBV, HCV or HIV infection	198 (14.4%)
Intranasal drug use	153 (11.13%)
Percutaneous/mucosal exposure to blood from an individual with HBV, HCV or HIV infection	20 (2.44%)*
Imprisonment	18 (1.31%)
Non-sterile tattoo or piercing	30 (2.18%)
Close contact of person infected with HBV or HCV	118 (8.58%)

*Data on Percutaneous/Mucosal Exposure to Blood From an Individual With HBV, HCV or HIV Infection was Gathered From 2017-2022, n=819.

Risk behaviours with increasing trends over the study period were history of injection drug use (IDU) (τ=0.51, p=0.049, *Q*_*i*_=1.07 [0.6-1.75]); men who had sex with men (MSM) (τ=0.71, p=0.005, *Q*_*i*_=0.68 [0.22-1.05]); history of sex in exchange for drugs or money, (τ=0.7, p=0.006, *Q*_*i*_=0.42 [0.2-0.63]); sex with a person from the previous risk groups (τ=0.58, p=0.02, *Q*_*i*_=1.23 [0.21-2.37]); and intranasal drug use (τ=0.64, p=0.012, *Q*_*i*_=1.63 [0.82-2.7]). Trends are depicted in Figure S1. Trend analysis for risk behaviours is reported in Table S2.

**Figure 3.**
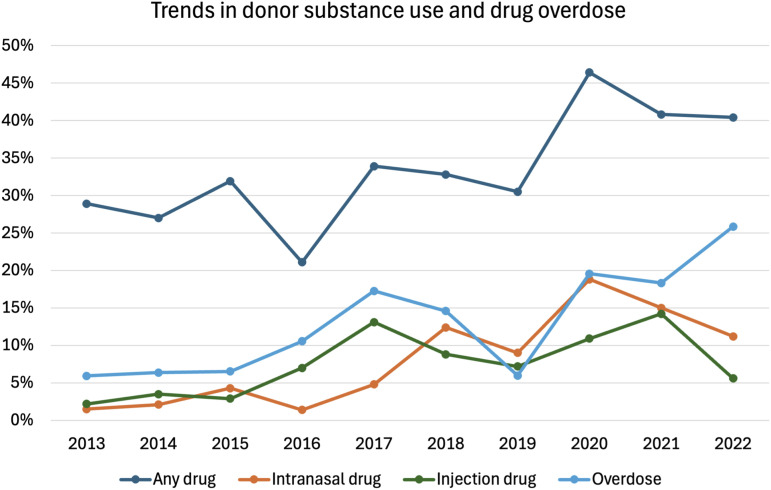
Percentage of drug use, intranasal drug use, injected drug use and death from overdose in organ donors from Alberta over the study period

There was an increase in overdose (τ=0.68, p=0.007, *Q*_*i*_=2.05 [0.46-2.76]) and cardiac arrest (τ=0.53, p=0.038, *Q*_*i*_=1.42 [0.11-2.57]) as causes of donor death over the study period. Figure 3 depicts overall donor drug use, intranasal drug use, IDU and overdose as a cause of death over the study period. Figure S2 depicts trends in causes of death. Trend analysis is summarized in Table S3.

Associations between donor risk behaviours and anti-HBc, anti-HCV and HCV NAT positivity are summarized in Table 3. Sex with an individual with suspected HBV, HCV, or HIV infection and being a close contact of an individual with HBV infection were the only risk behaviours associated with anti-HBc positivity; OR 11.97 (3.61-39.66) p<0.001 and OR 6.31 (2.29-17.33) p<0.001, respectively. We performed multivariate analysis; to avoid redundancy we combined the variables of sex with an individual with suspected HBV, HCV, or HIV infection and being a close contact of an individual with HBV infection. The final model included donor age, donor sex, and the combined variable of close contact and sex with someone with suspected HIV or viral hepatitis infection. Older donor age and close contact or sexual intercourse with a person suspected to have viral hepatitis were significantly associated with donor anti-HBc positivity: Donor age OR 1.05 (1.03-1.07) p<0.001; donor male sex OR 0.75 (0.41-1.37) p=0.41; donor with close contact or sexual intercourse with a person suspected to have viral hepatitis OR 8.37 (2.91-24.09) p<0.001.

**Table 3.** Association Between Behavioural Risk Factors and Positive Hepatitis B Core Antibody, Hepatitis C Virus Antibody, and Hepatitis C Virus Nucleic Acid Amplification test. Anti-HBc: Hepatitis B Core Antibody, Anti-HCV: Hepatitis C Virus Antibody. HBV: Hepatitis B Virus, HCV: Hepatitis C Virus, NAT: Nucleic Acid Amplification Test, OR: Odds Ratio

Variable	OR (95%CI) Anti-HBc	p	OR (95%CI) Anti-HCV	p	OR (95%CI) HCV NAT	p
Overdose	1.64 (0.78-3.46)	0.18	8.39 (4.72-14.91)	<0.001	6.48 (3.32-12.65)	<0.001
Any drug use	0.83 (0.44-1.56)	0.57	11.98 (6.34-51.2)	<0.001	18.03 (6.34-51.2)	<0.001
Intranasal drug use	1.06 (0.37-3.03)	0.9	3.05 (1.48-6.27)	0.002	2.85 (1.22-6.66)	<0.015
Non-injected, non-intranasal drug use	0.47 (0.18-1.2)	0.117	4.44 (1.73-11.3)	0.002	6.17 (1.79-21.26)	0.004
Injection drug use	1.42 (0.55-3.68)	0.46	28.86 (15.6-53.7)	<0.001	32.7 (15.8-67.6)	<0.001
Men who have sex with men	2.21 (0.50-9.8)	0.29	2.63 (0.76-9.12)	0.126	2.97 (0.65-13.4)	0.156
Sex in exchange for drugs or money	2.14 (0.63-7.19)	0.21	11.1 (5.22-23.59)	<0.001	8.43 (3.47-20.4)	<0.001
Sex with an individual with the previous 3 risk categories	2.38 (0.98-5.7)	0.55	16.2 (8.81-30)	<0.001	26.6 (12.2-49.3)	<0.001
Sex with an individual with suspected HBV, HCV or HIV infection	11.97 (3.61-39.66)	<0.001	80.48 (24.2-267.3)	0<0.001	44.3 (14.6-134.4)	<0.001
Percutaneous or mucosal exposure to blood from an individual with HBV, HCV or HIV infection	3.72 (0.81-17.05)	0.09	1.03 (0.13-7.96)	0.97	3.24 (0.71-14.76)	0.128
Imprisonment	1.06 (0.32-3.51)	0.91	7.94 (4.14-15.23)	<0.001	6.06 (4.07-14.18)	<0.001
Non-sterile tattoo or piercing	1.26 (0.16-9.56)	0.82	4.07 (1.17-14.18)	0.017	3.58 (0.8-15.88)	0.093
Close contact with an individual with HBV or HCV infection.	6.31 (2.29-17.33)	<0.001	23.6 (10.57-52.89)	<0.001	13.95 (5.53-35.1)	<0.001

Except for MSM or percutaneous/mucosal exposure to blood from an individual with HBV, HCV or HIV, most of the risk behaviours used to determine IRD status were significantly associated with positive donor anti-HCV and HCV NAT.

Any drug use and overdose as a cause of death were associated with HCV NAT positivity, OR 18.03 (6.34-51.2) p<0.001 and OR 6.48 (3.32-12.65) p<0.001, respectively. Non-injected/non-intranasal drug use remained strongly associated with HCV NAT positivity with an OR 6.17 (1.79-21.26) p=0.004. There was a gradient of association depending on drug administration route with IDU being the strongest, OR 32.76 (15.87-67.6) p<0.001.

## Discussion

We documented an increase in donors classified as IRD over the study period in Alberta. In Manitoba there was a trend that did not reach statistical significance, likely secondary to a lower number of donors. The increased proportion of IRD was driven by positive trends in several risk factors. The risk factor with the steepest slope was intranasal drug use, followed by history of sex with persons in other risk categories and injection drug use. Concordantly, drug overdose became a prevalent cause of donor death during the study period.

Our results highlight several challenges with IRD labeling in deceased organ donation in Canada. The regulatory framework does not address handling of “I don’t know” responses. Unless an OPO chooses to consider this as a positive response, there is no requirement for follow up NAT testing in the recipient. “I don’t know” responses in our study were particularly encountered in items inquiring about donor sexual behavior. Questions may be interpreted differently by next of kin. For example, MSM may be perceived as an identity-based criterion; that the patient was a Two-Spirit, Gay, Bisexual, Queer or other MSM (2S/GBQ+). Even though MSM is framed as a behavioral criterion next of kin may give a negative answer if the donor did not have an open 2S/GBQ+ identity.^7^ Previous work has highlighted the imprecision of next of kin interrogation regarding deceased donor risk factors.^8^

The prevalence of active hepatitis B (HBsAg positive) was low among organ donors in our cohort and may reflect the low prevalence of the disease in Canada. It could also be secondary to referral bias and exclusion of hepatitis B surface antigen positive individuals from deceased donation. In the Canadian setting risk of hepatitis B could be better captured by risk factors not included in the current donor IRD risk assessment such as age and country of birth.^9^ In our study age and close contact or sexual intercourse with an individual with suspected viral hepatitis were the only variables associated with anti-HBc positivity.

Most of the behavioural risk factors used to determine IRD status were found to be associated with donor HCV infection; only MSM and percutaneous or mucosal exposure to blood from an infected individual were not associated with HCV status. A greater prevalence of HCV infection has been documented among MSM living with HIV, but this is less pronounced when comparing MSM not living with HIV and general population.^10^ Recent studies have highlighted potential discriminatory practices regarding gender-diverse individuals access to organ donation.^11^ The lack of association of donor MSM with increased risk of donor bloodborne pathogen infection supports removing this criterion and moving to gender neutral sexual risk based questions.^7,12^

Multiple donor questionnaire items had a positive association with HCV infection; this is a positive feature of the screening procedure. However, it is important to mention that the risk behaviours used to determine IRD status have remained static despite shifting epidemiology of HCV and HIV in Canada. Hepatitis C infection was classically considered to be prevalent in people born between 1945-1965. It is now prevalent in communities with adverse social determinants of health, driven by increasing drug use and transactional sex.^10,13-15^ The screening questionnaire does not consider non-injected/non-intranasal drug as a risk factor for these infections. Our study found that use of non-intranasal/non-injected drugs was associated with HCV infection. Similarly, cause of death is not considered for adjudication or IRD status, our data show association between drug overdose and hepatitis C infection.^16^

The potential for missing bloodborne infections in traditional serologic screening assays was the concern that drove the creation of the IRD label. If a donor acquires an infection shortly before donation it may not be detected by serology.^17-19^ This led to introduction of more sensitive donor screening with NAT assays. Universal NAT screening for organ donors in Canada will be implemented by August 2026 (Regulatory communication, unpublished).

Previous studies reported lower rates of transplantation of IRD organs in Canada, and this was partially mitigated by NAT screening.^20^ Data from an Ontario transplant program reported a proportion of IRD of 18.6% in the period of 2013-2015.^21^ The proportion of IRD organs in the later years of our study hovered around 30%. This clear trend favors policy change to more sensitive screening methods. Serologic assays however also remain useful. In the context of hepatitis C as this may serve as a marker of previous exposure, but the disease does not generate protective immunity and reinfection is possible. Anti-HCV may be a marker for preexisting risk behavior. Hepatitis B serology is still necessary since isolated anti-HBc donors transmit the disease to liver recipients in the absence of prophylaxis.^22^ Despite enhanced sensitivity NAT testing can be negative during the short eclipse period when circulating viral genetic material is below the level of detection by current assays. Currently, there is no Canadian regulatory requirement planned to expand recipient NAT for HBV/HCV/HIV beyond recipients of IRD organs. Recent data from the US demonstrate the critical importance of universal recipient NAT to optimize safety.^23^ Despite universal NAT screening US guidelines mandate recipient post-transplant NAT testing regardless of donor risk profile.^24^ European Council guidelines on safety of organ transplantation similarly recommend universal recipient screening.^25^ Our data support Canadian policy review regarding universal recipient posttransplant NAT given that the IRD designation was demonstrated to incompletely capture HCV risk.

An important discussion is whether residual risk of donor infection when NAT screening is employed continues to warrant IRD designation. Risk of undetected infection when utilizing universal NAT testing is low. Modelling of “greatest risk” donors, meaning those engaged in highest behavioural risks up to the time of donation, suggest the risk is 34.7 per 10,000 for HIV, 13.4 per 10,000 for HBV and 27.6 per 10,000 for HCV^17^; HCV is now curable, and HBV and HIV are now treatable with highly effective and safe therapies. In the United States higher non-utilization rates for organs procured from donors labelled as IRD was demonstrated.^26^Non-utilization of organs from IRD donors results in worse recipient outcomes, including survival.^27,28^ The intense focus on risk behaviours and labelling itself may be stigmatizing for donor families and negatively impact donation consent.^12^ The lack of added value and negative impact on recipient outcomes led to elimination of the IRD label and adoption of universal donor and recipient NAT screening in the US in 2020.^24^

There are several limitations to this study. Its retrospective nature has the potential for bias. Donor assessment questionnaires are not standardized across jurisdictions; although all questionnaires must address the necessary regulatory elements of the history, the wording and order of these questions is up to the OPO. This precluded complete merging of data from Alberta and Manitoba. Importantly, the study only captured data from organ donors but not potential donors. Referral bias would not be captured by this data. Disease epidemiology may be variable across provinces and results may not be generalizable country wide, although our cohort accounts for 2 provinces and 20.6% of all organ donors in Canada during this time period.^29^ Finally, it is not possible to draw conclusions with regards to donor HIV risk. No donor had HIV infection.

In conclusion this study demonstrates an increasing rate of IRD donors. We documented an important proportion of “I don’t know” responses to the donor questionnaire and encountered variables not associated with infection risk (MSM, percutaneous exposure to body fluids). We found variables associated with donor bloodborne infection (cause of death and any drug use) that are not considered IRD criteria at the present time. This highlights the importance of understanding regional HBV/HCV/HIV transmission patterns when assessing donor risk. This information may also guide adjustments to donor risk assessment and policy discussions regarding universal recipient NAT and the utility of the IRD label in Canadian organ donation as universal donor NAT is implemented in 2026.

## Supplemental Material

Supplemental Material - Trends in Increased Infection Risk Deceased Organ Donors. A Two-Centre Retrospective Study Supplemental Material for Trends in Increased Infection Risk Deceased Organ Donors. A Two-Centre Retrospective Study by Oscar A. Fernández‐García, MD, Justin Co, MD, Tanya Suandork, BSc, Murdoch Leeies, MD, MSc, Karen Elizabeth Doucette, MD, MSc in Canadian Journal of Kidney Health and Disease

Supplemental Material - Trends in Increased Infection Risk Deceased Organ Donors. A Two-Centre Retrospective Study Supplemental Material for Trends in Increased Infection Risk Deceased Organ Donors. A Two-Centre Retrospective Study by Oscar A. Fernández‐García, MD, Justin Co, MD, Tanya Suandork, BSc, Murdoch Leeies, MD, MSc, Karen Elizabeth Doucette, MD, MSc in Canadian Journal of Kidney Health and Disease

Supplemental Material - Trends in Increased Infection Risk Deceased Organ Donors. A Two-Centre Retrospective Study Supplemental Material for Trends in Increased Infection Risk Deceased Organ Donors. A Two-Centre Retrospective Study by Oscar A. Fernández‐García, MD, Justin Co, MD, Tanya Suandork, BSc, Murdoch Leeies, MD, MSc, Karen Elizabeth Doucette, MD, MSc in Canadian Journal of Kidney Health and Disease

## Data Availability

The datasets generated during and/or analyzed during the current study are not publicly available due organ donor confidentiality but are available from the corresponding author on request.*

## Appendix

### Abbreviations

Ab: Antibody
Anti-HBc: Hepatitis B core antibody
Anti-HCV: Hepatitis C virus antibody
CSA: Canadian Standards Association
CTO: Cells tissues and organs
HBV: Hepatitis B virus
HBsAg: Hepatitis B virus surface antigen
HCV: Hepatitis C virus
HIV: Human immunodeficiency virus
IDU: Injection drug use
IRD: Increased risk donor
MSM: Men who have sex with men
NAT: Nucleic acid amplification test
OPO: Organ procurement organization
SOT: Solid organ transplant
SOTR: Solid organ transplant recipient.
