# Supplementary material for: Trends in Increased Infection Risk Deceased Organ Donors. A Two-Centre Retrospective Study
Source: Can J Kidney Health Dis. 2026 Jun 24;13:20543581261463456. doi: 10.1177/20543581261463456 (PMC13305771; doi:10.1177/20543581261463456)
Supplement: Supplemental Material - Trends in Increased Infection Risk Deceased Organ Donors. A Two-Centre Retrospective Study [file sj-pdf-1-cjk-10.1177_20543581261463456.pdf]

CJKHD-26-0010.R1

Trends in Increased Infection Risk Deceased Organ Donors. A Two-Centre Retrospective Study

Résumé:

Contexte:

Au Canada, les normes réglementaires exigent que la classification « donneur à risque accru » (DRA) soit apposée aux donneurs présentant des facteurs de risque associés à l'hépatite B (VHB), à l'hépatite C (VHC) et aux infections par le virus de l'immunodéficience humaine (VIH). Cette étude visait à évaluer les tendances temporelles du taux de DRA et des facteurs de risque associés, ainsi que leur lien avec les résultats de dépistage du VHB, du VHC et du VIH.

Méthodologie :

Étude rétrospective portant sur des donneurs d'organes décédés dans deux centres canadiens (University of Alberta Hospital et Transplant Manitoba) sur une période chevauchante allant de 2013 à 2022. La proportion de dons provenant de DRA au fil du temps a été évaluée au moyen d'une analyse des tendances. Ont également été explorées la tendance des facteurs de risque et leur association avec les résultats du dépistage.

Résultats :

Des 1 491 donneurs recensés, 332 (22,3 %) étaient des DRA. Une augmentation significative des DRA a été observée en Alberta. Au cours de la période étudiée, la consommation de drogues (RC : 18,03 [6,34 à 51,2]; $p < 0,001$) et les décès par surdose (RC : 6,48 [3,32 à 12,65]; $p < 0,001$) étaient en hausse significative

chez les donneurs et ont été fortement corrélés à la présence d’une infection par le VHC. Les donneurs classés HARSAH (hommes ayant eu des relations sexuelles avec des hommes) n’ont pas présenté un risque accru de contracter le VHB, le VHC ou le VIH. Certaines questions de l’évaluation des facteurs de risque chez les donneurs présentaient jusqu’à 20 % de réponses « je ne sais pas ».

Conclusion :

Notre étude démontre qu’une proportion croissante des dons d’organes provient de DRA dans deux centres de transplantation canadiens. Les exigences réglementaires comprennent des variables qui ne sont pas associées au risque d’infection par le VHB, le VHC ou le VIH, tout en omettant certains facteurs associés à ce risque dans cette cohorte. La proportion de réponses « je ne sais pas » à l’évaluation des risques pour les donneurs met en évidence ses limites. Ces données pourraient éclairer l’évaluation des risques pour les donneurs dans le contexte canadien et mettre en évidence l’évolution des profils épidémiologiques du VHB, du VHC et du VIH chez les donneurs d’organes.
