# Supplementary material for: Trends in Increased Infection Risk Deceased Organ Donors. A Two-Centre Retrospective Study
Source: Can J Kidney Health Dis. 2026 Jun 24;13:20543581261463456. doi: 10.1177/20543581261463456 (PMC13305771; doi:10.1177/20543581261463456)
Supplement: Supplemental Material - Trends in Increased Infection Risk Deceased Organ Donors. A Two-Centre Retrospective Study [file sj-pdf-2-cjk-10.1177_20543581261463456.pdf]

Supplementary material

Supplementary Table 1: Canadian Standards Association Annex E. Factors and behaviours associated with a higher risk of human immunodeficiency virus (HIV), hepatitis B virus (HBV), and hepatitis C virus (HCV)

Assessment of donors 11 years of age or older

The assessment of donors 11 years of age or older shall include the following risk factors and risk behaviours associated with HIV, HBV, and HCV:

a) Persons who report non-medical intravenous, intramuscular, or subcutaneous infection of drugs in the preceding 12 months;
b) Men who have had sex with another man in the preceding 12 months;
c) Persons who have engaged in sex in exchange for money or drugs in the preceding 12 months;
d) Persons who have had sex in the preceding 12 months with any persons described in Items a) to c) or with a person known or suspected to have HIV, or clinically active HBV or clinically active HCV;
e) Persons with a history of intranasal drug use for non-medical reasons in the last 6 months, unless HCV NAT is performed and found to be negative;
f) Persons who have been exposed, in the preceding 12 months to known or suspected HIV, HBV and/or HCV-infected blood through percutaneous inoculation or through contact with an open wound, nonintact skin, or mucous membrane;
g) Persons who have been in a youth correctional facility, jail, or prison for more than 72 consecutive hours in the preceding 12 months;
h) Persons who within 12 months preceding donation have undergone tattooing, ear piercing, or body piercing in which sterile procedures were not used (e.g., contaminated instruments and/or ink were used, or shared instruments that had not been sterilized between uses were used); and
i) Persons who have had close contact within 12 months preceding donation with another person having clinically active HBV or clinically active HCV infection (e.g., living in the same household, where sharing of kitchen and bathroom facilities occurs regularly.

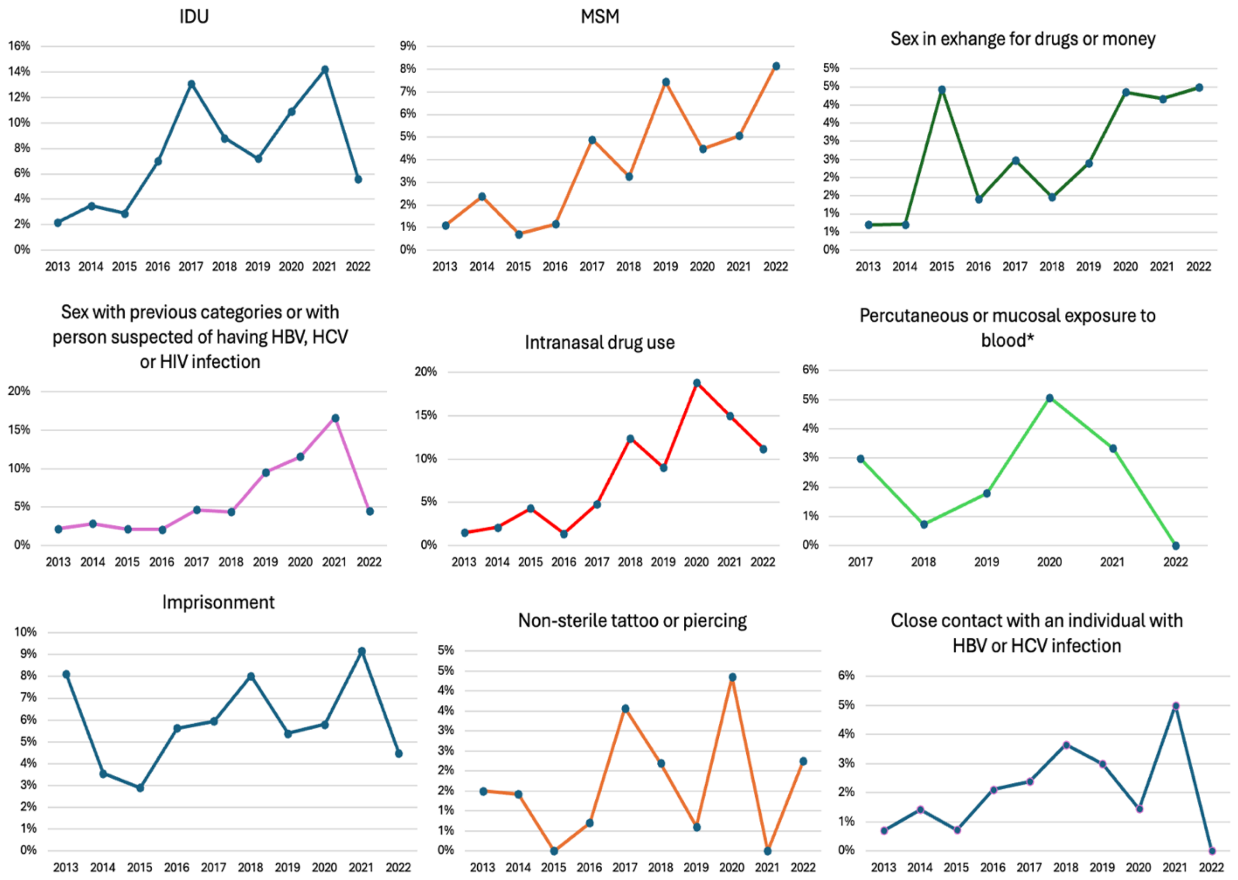

Supplementary figure 1. Donor behavioural risk factors for blood-borne viral infections study period in Alberta. IDU: Injection drug use. MSM: Men who have sex with men. *Percutaneous exposure to blood was recorded from 2017 onwards.

Risk behaviour	MK τ	MK p	Sen's slope	Sen's slope 95% CI

IVDU	0.51	0.049	1.075	0.06, 1.75
MSM	0.71	0.005	0.68	0.22, 1.05
Sex in exchange for drugs of money	0.7	0.006	0.42	0.2, 0.63
Sex with the above categories	0.58	0.024	1.23	0.21, 2.37
Intranasal drug use	0.64	0.012	1.63	0.82, 2.7
Percutaneous or mucosal exposure to HBV, HCV, HIV	0.09	0.879	0.07	-1.8, 1.1
Imprisonment	0.11	0.72	0.13	-0.4, 0.81
Nonsterile tattoo or piercing	0.06	0.85	0.07	-0.28, 0.53
Close contact with person infected with HBV or HCV	0.29	0.279	0.32	-0.17, 0.58
Any drug use*	0.51	0.049	1.48	0.26, 3.21

Supplementary table 2. Trend analysis for yearly percentage of donor risk behaviours for infection with HBV, HCV and HIV between 2013-2022, in Alberta.

MK: Mann-Kendall test, CI: Confidence interval, IVDU: Intravenous drug use, MSM: Men who have sex with men, HBV: Hepatitis B virus, HCV: Hepatitis C virus, HIV: Human immunodeficiency virus. * Any drug use is currently not an IRD classification criterion.

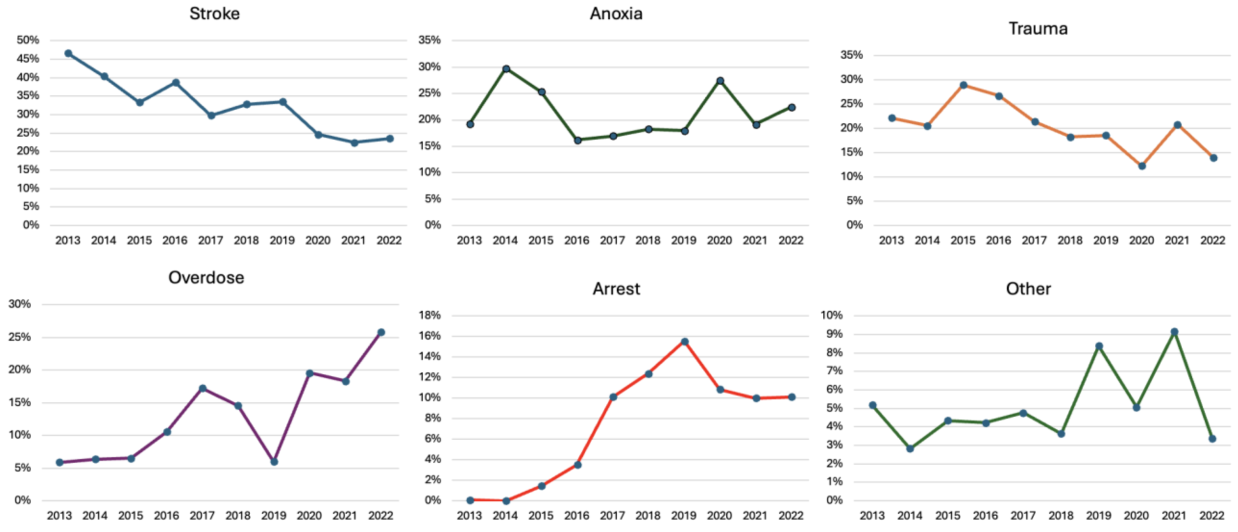

Supplementary figure 2. Donor causes of death over study period in Alberta. HBV: Hepatitis B virus, HCV: Hepatitis C virus, HIV: Human immunodeficiency virus.

Cause of death	MK τ	MK p	Sen's slope	Sen's slope 95% CI
Stroke	-0.73	0.004	-2.31	-3.3, -1.39
Anoxia	0.02	1	0.3	-1.51, 1.05
Trauma	-0.51	0.049	-1.35	-2.72, -0.14
Overdose	0.68	0.007	2.05	0.46, 2.76
Cardiac arrest	0.53	0.038	1.42	0.11, 2.57
Other	0.15	0.59	0.2	-0.23, 0.8

Supplementary table 3. Trend analysis for yearly percentage of donor causes of death between 2012-2023 in Alberta.

MK: Mann-Kendall test, CI: Confidence interval
