# Supplementary material for: Trends in Increased Infection Risk Deceased Organ Donors. A Two-Centre Retrospective Study
Source: Can J Kidney Health Dis. 2026 Jun 24;13:20543581261463456. doi: 10.1177/20543581261463456 (PMC13305771; doi:10.1177/20543581261463456)
Supplement: Supplemental Material - Trends in Increased Infection Risk Deceased Organ Donors. A Two-Centre Retrospective Study [file sj-pdf-3-cjk-10.1177_20543581261463456.pdf]

STROBE Statement—checklist of items that should be included in reports of observational studies

	Item No	Recommendation
**Title and abstract**	1	Indicate the study's design with a commonly used term in the title or the abstract<br>Page 1: Title specifies that this is a two-centre retrospective study.
		(*b*) Provide in the abstract an informative and balanced summary of what was done and what was found<br>Page 3: Abstract reports the main findings of the study.
**Introduction**		
Background/rationale	2	Explain the scientific background and rationale for the investigation being reported<br>Page 4, background explains the Canadian framework for deceased donor risk assessment. The project's aim is mentioned on page 5:<br>The aim of this project was to describe and analyse trends regarding deceased donor risk factors for bloodborne infections to inform evidence-based policy.<br>This is followed by the primary and secondary outcomes.
Objectives	3	State specific objectives, including any prespecified hypotheses<br>Primary and secondary outcomes are clearly described on page 6.
**Methods**		
Study design	4	Present key elements of study design early in the paper<br>Page 6 & 7. Methods section<br>There is a clear description of the origin of the datasets along with the time period of the respective datasets. Inclusion and exclusion criteria are outlined.
Setting	5	Describe the setting, locations, and relevant dates, including periods of recruitment, exposure, follow-up, and data collection<br>Page 6.<br>Data sets comprised deceased organ donors who donated organs at the University of Alberta Hospital multiorgan transplant program between January 1, 2013, and December 31, 2022. Through a previous project, the Authors had access to a convenience dataset of deceased organ donors from Manitoba encompassing the years 2015-2020.
Participants	6	(*a*) *Cohort study*—Give the eligibility criteria, and the sources and methods of selection of participants. Describe methods of follow-up<br>Page 6: "Deceased organ donors who donated organs during the specified periods were included; living donors were excluded."<br>Data sources mentioned in page 5: "Donor charts from Give Life Alberta and Transplant Manitoba, the organ procurement organizations (OPO) serving their respective transplant programs were reviewed."<br>*Case-control study*—Give the eligibility criteria, and the sources and methods of case ascertainment and control selection. Give the rationale for the choice of cases and controls<br>*Cross-sectional study*—Give the eligibility criteria, and the sources and methods of selection of participants
		(*b*) *Cohort study*—For matched studies, give matching criteria and number of exposed and unexposed

		Case-control study—For matched studies, give matching criteria and the number of controls per case
Variables	7	Clearly define all outcomes, exposures, predictors, potential confounders, and effect modifiers. Give diagnostic criteria, if applicable<br>Page 5: "Cause of death, responses to questionnaire items and donor screening results were recorded."
Data sources/ measurement	8*	For each variable of interest, give sources of data and details of methods of assessment (measurement). Describe comparability of assessment methods if there is more than one group<br>Page 6: "Donor charts from Give Life Alberta and Transplant Manitoba, the organ procurement organizations (OPO) serving their respective transplant programs were reviewed."<br>Page 6 & 7: Cause of death, responses to questionnaire items and donor viral screening results were recorded. Donor IRD status was classified based on responses to questionnaire items addressing the risk factors outlined in Annex E of the Canadian Standards Association (CSA) Cells, Tissues, and Organs for Transplant: General requirements. Different coding of risk factors at collection by clinical teams precluded complete merging of the two cohorts. The donor cohort from University of Alberta was used to explore the secondary outcomes
Bias	9	Describe any efforts to address potential sources of bias<br>Page 6: Missing data consisted of "I don't know" responses and was analysed as a secondary outcome.<br>Page 7: Different coding of risk factors at collection by clinical teams precluded complete merging of the two cohorts. The donor cohort from University of Alberta was used to explore the secondary objectives.
Study size	10	Explain how the study size was arrived at<br>This is not an interventional study, sample size calculation was not performed.
Quantitative variables	11	Explain how quantitative variables were handled in the analyses. If applicable, describe which groupings were chosen and why<br>Page 7: "Categorical variables were expressed as percentages; continuous variables were expressed as median and interquartile range.
Statistical methods	12	(*a*) Describe all statistical methods, including those used to control for confounding<br>Page 7: Trends in proportion of IRD donors are presented for both centres. Mann-Kendall test and Sen's slope were used for trend analysis.<br>Odds ratios for HBV and HCV donor infection were calculated using bivariate logistic regression. Given that very few donors had detectable HBV viral load or a positive hepatitis B surface antigen (HBsAg), HBV analysis was limited to association with antibody to hepatitis B core antigen (anti-HBc). When performing multivariate analysis, we used logistic regression with backwards elimination of variables to determine the final variables to be included in the model. Variables with p>0.10 were eliminated unless required for adjustment (age and sex).
		(*b*) Describe any methods used to examine subgroups and interactions<br>See above.
		(*c*) Explain how missing data were addressed<br>Missing data consisted of "I don't know" responses and was analysed as a secondary outcome.
		(*d*) *Cohort study*—If applicable, explain how loss to follow-up was addressed<br>All subjects were deceased donors so there was no follow-up.<br>*Case-control study*—If applicable, explain how matching of cases and controls was addressed

	Cross-sectional study—If applicable, describe analytical methods taking account of sampling strategy
	(*e*) Describe any sensitivity analyses

Results

Participants	13*	(a) Report numbers of individuals at each stage of study—eg numbers potentially eligible, examined for eligibility, confirmed eligible, included in the study, completing follow-up, and analysed<br>All organ donors during the study period were included.
		(b) Give reasons for non-participation at each stage<br>All donors were included, not applicable.
		(c) Consider use of a flow diagram.<br>All donors were included, not applicable.
Descriptive data	14*	(a) Give characteristics of study participants (eg demographic, clinical, social) and information on exposures and potential confounders<br>Page 8 and Table 1. During the study period 1375 deceased donors donated to the University of Alberta Hospital and 116 to the Manitoba Transplant programs. Donor characteristics and prevalence of HBV, HCV and HIV infection are shown in **Table 1.**
		(b) Indicate number of participants with missing data for each variable of interest<br>There was no missing data given nature of the variables (cause of death) and transplant regulations ensuring donor risk assessment and screening.
		(c) *Cohort study*—Summarise follow-up time (eg, average and total amount)<br>Subjects were not followed over time.
Outcome data	15*	*Cohort study*—Report numbers of outcome events or summary measures over time<br>Page 8, figures 1 & 2.<br>Increased risk donors accounted for 40% of ED organs in Alberta and 44% in Manitoba.
		Case-control study—Report numbers in each exposure category, or summary measures of exposure
		Cross-sectional study—Report numbers of outcome events or summary measures
Main results	16	Give unadjusted estimates and, if applicable, confounder-adjusted estimates and their precision (eg, 95% confidence interval). Make clear which confounders were adjusted for and why they were included<br>Page 9. Confidence intervals are consistently reported for measures regarding trend analysis and logistic regression. "Man-Kendall test demonstrated a significant increasing trend (Tau=0.56, p=0.0304, Sen's slope: Q_i=2.6 [0.6-4.25]) of IRD donors in Alberta. An increasing proportion of IRD donors was noted in Manitoba however the trend was not statistically significant (Tau=0.73, p=0.0602, Q_i=6 [-3.5 to 18])."
		(*b*) Report category boundaries when continuous variables were categorized
		(*c*) If relevant, consider translating estimates of relative risk into absolute risk for a meaningful time period
Other analyses	17	Report other analyses done—eg analyses of subgroups and interactions, and sensitivity analyses<br>Page 9-12 and supplementary material.<br>Risk behaviours with increasing trends over the study period were history of injection drug use (IDU) (Tau=0.51, p=0.049, Q_i=1.07 [0.6-1.75]); men who had sex with men (MSM) (Tau=0.71, p=0.005, Q_i=0.68 [0.22-1.05]); history of sex in exchange for drugs or money, (□=0.7, p=0.006, Q_i=0.42 [0.2-0.63]); sex with a person from the previous risk groups or

		suspected to be infected with a bloodborne virus (□=0.58, p=0.02, Q_i=1.23 [0.21-2.37]); and intranasal drug use (□=0.64, p=0.012, Q_i=1.63 [0.82-2.7]). Trends are depicted in **Supplementary Figure 1**. Trend analysis for risk behaviours is reported in **Supplementary Table 2**.<br>There was an increase in overdose (□=0.68, p=0.007, Q_i=2.05) and cardiac arrest (□=0.53, p=0.038, Q_i=1.42) as causes of donor death over the study period. **Figure 3** depicts overall donor drug use, intranasal drug use, IDU and overdose as a cause of death over the study period. Donor causes of death are depicted in **Supplementary Figure 2.** Trend analysis is summarized in **Supplementary Table 3**.<br>Associations between donor risk behaviours and anti-HBc, anti-HCV and HCV NAT positivity are summarized in **Supplementary Table 4**. Sex with an individual with suspected HBV, HCV, or HIV infection and being a close contact of an individual with HBV infection were the only risk behaviours associated with anti-HBc positivity; OR 11.97 (3.61-39.66) p<0.001 and OR 6.31 (2.29-17.33) p<0.001, respectively.
**Discussion**		
Key results	18	Summarise key results with reference to study objectives<br>Page 7<br>We documented an increase in donors classified as IRD over the study period in Alberta. In Manitoba the trend did not reach statistical significance, likely secondary to limited power given a lower number of donors. The increased proportion of IRD was driven by positive trends in several risk factors. The risk factor with the steepest slope was intranasal drug use, followed by history of sex with persons in other risk categories and injection drug use. Concordantly, drug overdose became a prevalent cause of donor death during the study period.
Limitations	19	Discuss limitations of the study, taking into account sources of potential bias or imprecision. Discuss both direction and magnitude of any potential bias<br>Page 9<br>There are several limitations to this study. Its retrospective nature has the potential for bias. Donor assessment questionnaires are not standardized across jurisdictions; although all questionnaires must address the necessary regulatory elements of the history, the wording and order of these questions is up to the OPO. This precluded complete merging of data from Alberta and Manitoba. The study only captured data from organ donors but not potential donors. Referral bias would not be captured by this data. Finally, it is not possible to draw conclusions with regards to donor HIV risk. No donor had HIV infection
Interpretation	20	Give a cautious overall interpretation of results considering objectives, limitations, multiplicity of analyses, results from similar studies, and other relevant evidence<br>Page 9<br>In conclusion this study demonstrates an increasing rate of IRD donors. We documented an important proportion of "I don't know" responses to the donor questionnaire and encountered variables not associated with infection risk (MSM, percutaneous exposure to body fluids). We found variables associated with donor bloodborne infection (cause of death and any drug use) that are not considered IRD criteria at the present time. Disease epidemiology may be variable across provinces and results may not be generalizable country wide.
Generalisability	21	Discuss the generalisability (external validity) of the study results

		Page 10: “Disease epidemiology may be variable across provinces and results may not be generalizable country wide.”
**Other information**		
Funding	22	Give the source of funding and the role of the funders for the present study and, if applicable, for the original study on which the present article is based<br>Page 1: Stated in the title page.

*Give information separately for cases and controls in case-control studies and, if applicable, for exposed and unexposed groups in cohort and cross-sectional studies.

Note: An Explanation and Elaboration article discusses each checklist item and gives methodological background and published examples of transparent reporting. The STROBE checklist is best used in conjunction with this article (freely available on the Web sites of PLoS Medicine at http://www.plosmedicine.org/, Annals of Internal Medicine at http://www.annals.org/, and Epidemiology at http://www.epidem.com/). Information on the STROBE Initiative is available at www.strobe-statement.org.
